# Birth mode is associated with layer-specific mechanical changes in fetal membranes

**DOI:** 10.1038/s41598-025-04752-4

**Published:** 2025-06-20

**Authors:** Philip Friedrich, Hanna Grubitzsch, Benjamin Wolf, Hannah M. Eichholz, Cary Tutmarc, Pablo Gottheil, Frank Sauer, Alissa Cornelis, Anne-Sophie Wegscheider, Bahriye Aktas, Josef A. Käs, Holger Stepan

**Affiliations:** 1 Peter Debye Institute for Soft Matter Physics, Leipzig University, 04103 Leipzig, Germany; 2 Department of Obstetrics, University Hospital Leipzig, 04103 Leipzig, Germany; 3 Department of Gynecology, University Hospital Leipzig, 04103 Leipzig, Germany; 4 Leipzig Institute for Meteorology, Leipzig University, 04103 Leipzig, Germany; 5 Center for Scalable Data Analytics and Artificial Intelligence, Leipzig University, 04105 Leipzig, Germany; 6 Department of Radiology, Charité - Universitätsmedizin Berlin, 10117 Berlin, Germany; 7 Institute for Histology, Cytology and Molecular Diagnostics, MVZ Prof. Dr. Med. A. Niendorf Pathologie Hamburg-West GmbH, 22767 Hamburg, Germany

**Keywords:** Fetal membranes, Rupture of membranes, Tissue viscoelasticity, Atomic force microscopy, Magnetic resonance elastography, Shear rheology, Biophysics, Soft materials, Medical research, Biological physics

## Abstract

Rupture of fetal membranes and subsequent full-term birth are prerequisites for neonatal health, and a preterm rupture can lead to life-threatening complications. Our study determines the mechanical properties of term fetal membranes to identify perinatal structural changes by a unique biophysical multiscale approach, including atomic force microscopy, shear rheology, tabletop magnetic resonance elastography (MRE), and high-resolution optical microscopy. Fetal membranes from term spontaneous vaginal deliveries were compared to those from primary cesarean sections, used as a control group for pre-labor membranes. Spontaneously delivered term fetal membranes are softer and easier to deform in MRE experiments (median stiffness: 1.9 kPa, IQR 1.6–2.4) compared to controls (4.7 kPa, IQR 3.8–5.6); *p* < 0.001) and show increased water diffusion (median: 1.78 × 10^−3^ mm^2^/s, IQR: (1.65–1.84) × 10^−3^ vs. 1.66 × 10^−3^ mm^2^/s, IQR (1.60–1.73) × 10^−3^; *p* = 0.047). Their intermediate connective tissue layer (i.e. the collagen-rich area enclosed by the amnion and chorion) exhibits less ordered fiber alignment (median order parameter: 0.52, IQR 0.44–0.58 vs. 0.55, IQR 0.47–0.62; *p* = 0.04) and a looser fiber structure, as indicated by a significantly lower fiber area fraction (median: 0.33, IQR 0.25–0.46 vs. 0.73, IQR 0.63–0.88; *p* < 0.001) compared to the control membranes. These layer-specific changes in both structure and viscoelasticity are evidence for the dominant role of the intermediate connective tissue in maintaining membrane stability and the onset of rupture. Our mechanical and histopathological findings highlight the potential of mechanics-based screening-methods to assess the risk of preterm rupture and preterm birth to reduce neonatal morbidity.

## Introduction

The human amniotic and chorionic membranes, collectively referred to as fetal membranes (FMs), are critical for maintaining pregnancy as they carry the intrauterine fetus and the amniotic fluid. Their integrity and full-term rupture, typically occurring at the onset of labor, are crucial for both maternal and fetal health^1–4^. Preterm premature rupture of membranes (PPROM), i.e. rupture before 37 weeks of gestation, affects about 3% of all pregnancies^5^. It is a leading cause of preterm births, which are often associated with various neonatal complications^6–10^. While certain risk factors such as a history of cervical conization, short cervical length and infections have been identified^11–17^, effective prevention and treatment of PPROM remain challenging, as many underlying structural, biochemical, and especially mechanical processes in humans are still poorly understood.

**Fig. 1:**
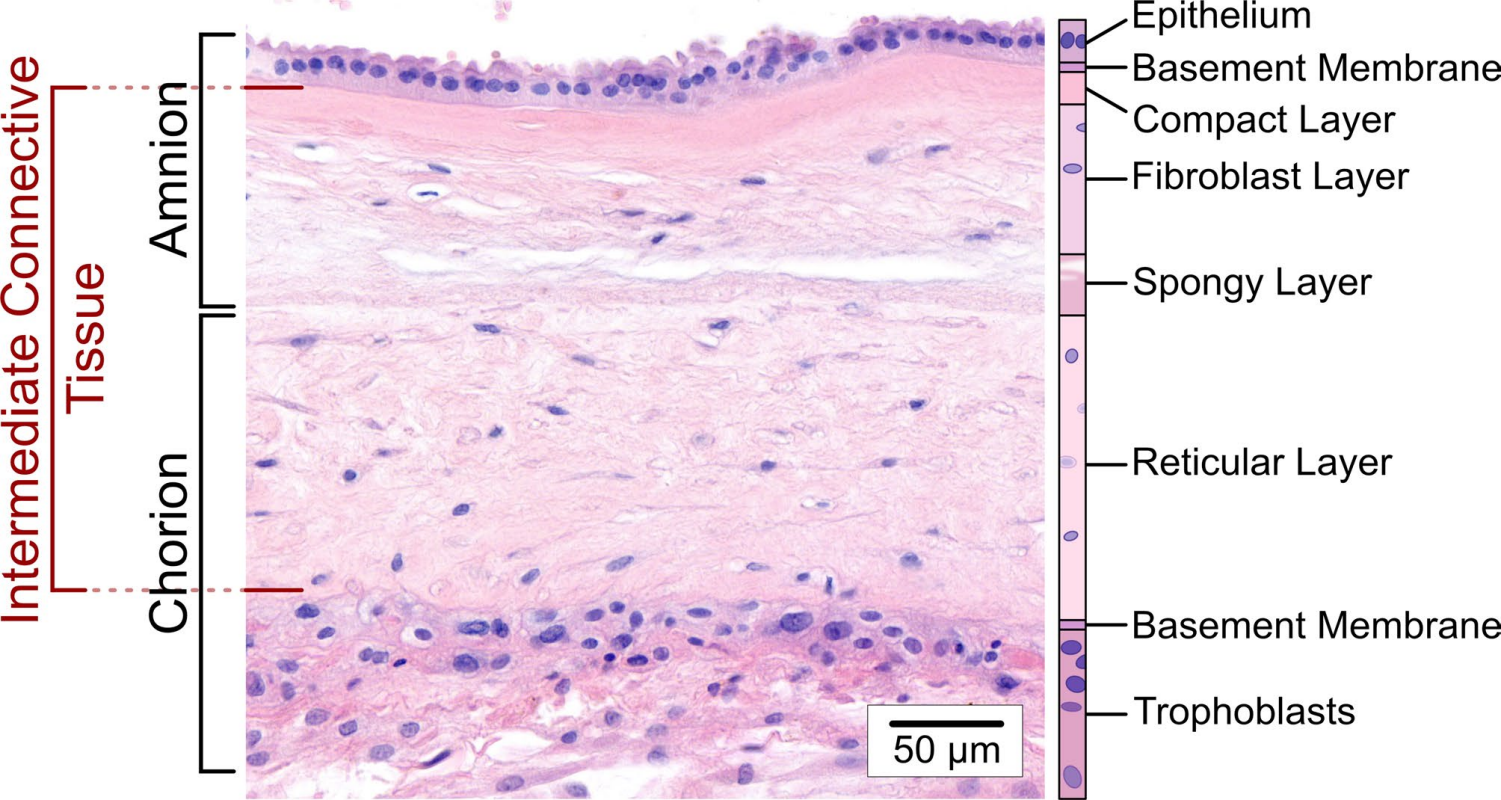
H&E-stained image section of the fetal membranes (FMs) obtained from a delivery via primary cesarean section showing the individual layers of amnion and chorion. The amniotic membrane consists of five sublayers: (1) a single-layered epithelium; (2) a thin basement membrane containing collagen types IV and VI along with glycoproteins; (3) a compact layer with densely packed collagen types I and III; (4) a fibroblast layer containing collagen types I and III, glycoproteins, fibroblasts, amniotic mesenchymal cells, and macrophages; and (5) a spongy layer rich in collagen type III^20^. The spongy layer enables dynamic coupling between amnion and chorion and is also referred to as the intermediate layer^64,65^. The outer layer of the FMs is the chorion, which connects them to the uterine cavity and the decidua (gestationally modified endometrium). It consists of three sublayers^66^: (1) a reticular layer containing fibrillar collagen types I, III, IV, V and VI and chorionic mesenchymal cells; (2) a basement membrane rich in collagen type IV and glycoproteins; and (3) a layer of chorionic trophoblast cells in contact with the trophoblast^32,67^. The two basement membranes of amnion and chorion surround several sublayers of collagen-rich extracellular matrix (ECM), which are here referred to as the intermediate connective tissue (ICT) of the FMs.

FMs are structurally complex, consisting of two primary layers: the amnion and the chorion. The amnion is a thin, avascular layer in direct contact with the embryo and is generally considered to dominate the mechanical properties of intact FMs^18–21^, while the chorion connects them to the uterine wall^22^. Together, they form a composite material consisting of several sublayers (see Fig. 1). The two basement membranes of amnion and chorion surround several sublayers of collagen-rich extracellular matrix (ECM) that contain few mesenchymal cells. These sublayers, which are referred to in the following as intermediate connective tissue (ICT), contribute to the structural integrity of the FMs throughout pregnancy^18,23–26^. Given this complex structure, the mechanical behavior of FMs is closely linked to their composition and organization.

While research has identified key molecular mechanisms involved in labor initiation, such as shifts in inflammation and matrix degradation^27–30^, the mechanical changes that underlie labor and membrane rupture remain poorly characterized. The tensile strength of FMs follows a triphasic curve^31^ and their stability depends on the collagen content and crosslinking^32^. However, previous studies primarily focused on biochemical aspects, leaving a critical gap in understanding the mechanical changes associated with labor and membrane rupture. Detailed layer-specific analyses remain rare. This gap in knowledge limits our ability to predict and mitigate the risk of preterm rupture, a condition with significant implications for neonatal health.

In this study, we address this gap by investigating the mechanical properties of term FMs directly after birth, comparing membranes from spontaneous deliveries (SD) with those from primary cesarean sections (CS) as a reference. These comparisons are crucial to understand how mechanical processes contribute to the initiation of labor and membrane rupture. By using advanced techniques previously applied to single cells, polymer networks, and primary tissues^33–39^, we quantify the viscoelastic properties of FMs at multiple length and time scales. This comprehensive approach highlights parturition-induced, layer-specific changes in mechanical behavior, with a focus on the ICT as a key structural component. Our findings, further supported by histological analyses of ECM fiber orientation and tissue integrity, reveal significant differences between both delivery types. These results provide valuable insights that may help to develop new mechanics-based screening methods to predict PPROM risk, ultimately contributing to reducing preterm birth and neonatal morbidity.

## Results

### FMs from spontaneous deliveries are mechanically softer in MRE experiments and show enhanced water diffusion

**Fig. 2:**
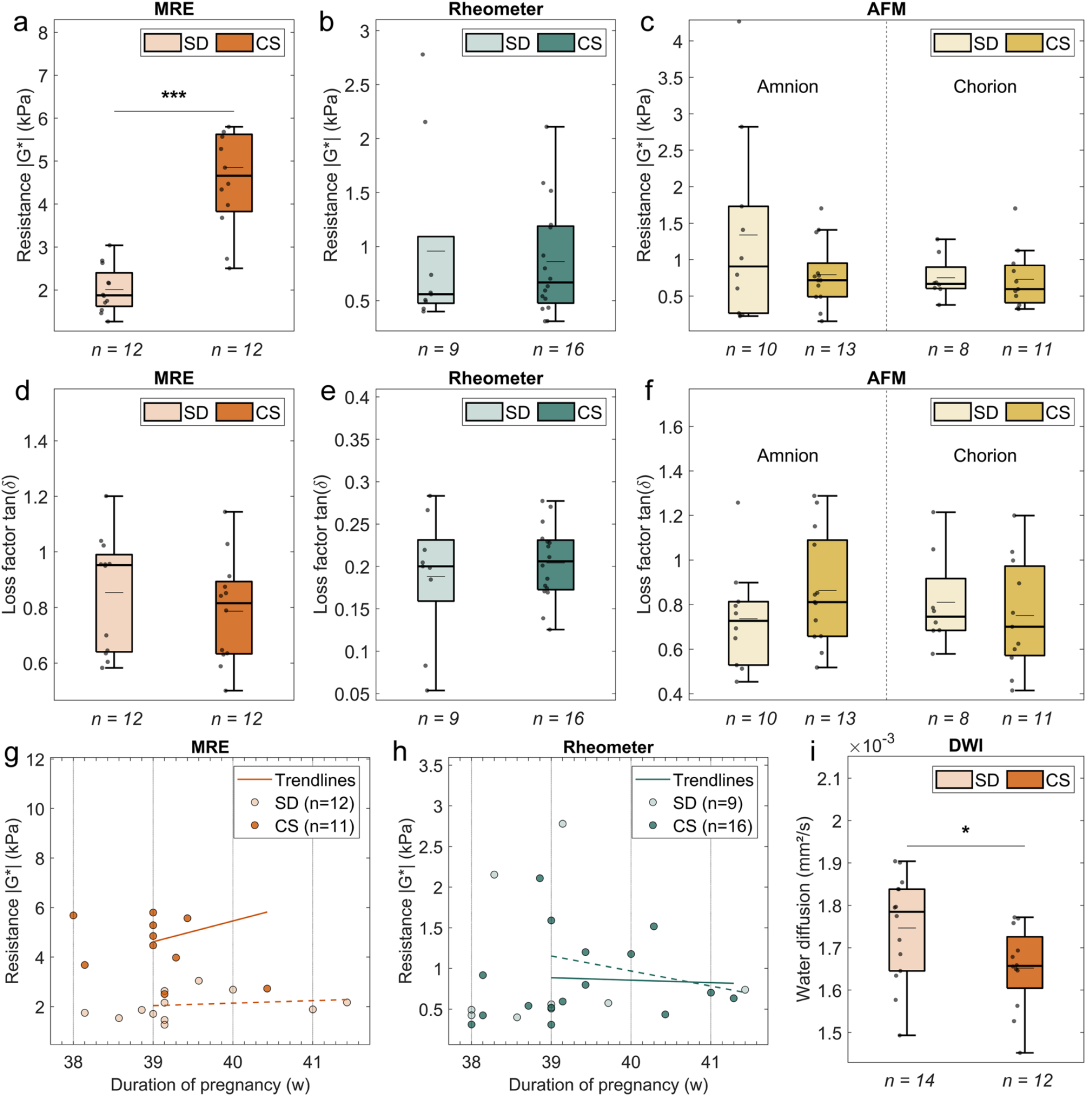
Mechanical properties and water diffusion of fetal membranes (FMs) collected from spontaneous vaginal deliveries (SD) and primary cesarean sections (CS). (**a**–**f**) Mechanical resistance (**a**–**c**) and energy dissipation (**d**–**f**) measured by MRE (**a**,**d**), rheometer (**b**,**e**), and AFM (**c**,**f**). The mechanical resistance is calculated from multi-frequency measurements as the magnitude of the complex shear modulus ($|G^{*} |$), and the energy dissipation as the loss factor (see Eq. 5 in Materials and Methods—Tabletop Magnetic Resonance Elastography). The results are shown at suitable frequencies (MRE: 1 kHz, rheometer: 1 Hz, AFM: 100 Hz). Significant differences between delivery types were observed only in MRE measurements: SD FMs show lower mechanical resistance than CS FMs (**a**, 1.9 kPa (IQR 1.6–2.4) vs. 4.7 kPa (IQR 3.8–5.6), *p* < 0.001). No significant differences were observed for rheometer (*p* = 0.76) or AFM measurements (amnion: *p* = 0.44; chorion: *p* = 0.54). In addition, the median loss factor shows a slightly higher value for SD FMs in MRE experiments (**d**, 0.95 (IQR 0.64–0.99) vs. 0.82 (IQR 0.63–0.89), *p* = 0.37), but is non-significant in rheometer (*p* = 0.80) or AFM experiments (amnion: *p* = 0.20; chorion: *p* = 0.44). (**g**–**h**) Scatter plots of mechanical resistance vs. pregnancy duration, measured by MRE (**g**) and rheometer (**h**), to assess stability trends as pregnancy reaches full term. Trendlines beginning at the end of the 39th week of pregnancy (i.e. at day 273) were added to the data, as this is the onset of decreasing tensile strength as reported by Pressman et al.^31^. No clear correlation with pregnancy duration was observed for SD (empty markers, dashed line) or CS (filled markers, solid line) FMs (**g**, MRE: SD: R^2^ = 0.024, CS: R^2^ = 0.055; h, rheometer: SD: R^2^ = 0.038, CS: R^2^ = 0.003). (**i**) Water diffusion, assessed via MRI as the apparent diffusion coefficient, shows a significant tendency towards higher values in SD FMs compared to CS FMs (1.78 × 10^−3^ mm^2^/s (IQR 1.65–1.84 × 10^−3^) vs. 1.66 × 10^−3^ mm^2^/s (IQR 1.60–1.73 × 10^−3^), *p* = 0.047). In the boxplots, individual samples are shown as dots, median (mean) values are represented as thick (thin) horizontal lines (*n* = number of samples). Statistical significance was determined using a Wilcoxon rank-sum test: *p < 0.05, **p < 0.01, ***p < 0.001.

FMs from 12 spontaneous vaginal deliveries (SD) and 12 primary cesarean sections (CS) were measured by MRE to characterize their overall viscoelastic bulk behavior. MRE assesses bulk tissue mechanics by inducing acoustic shear waves throughout the sample at different frequencies. The mechanical resistance (see Fig. 2a), described by the magnitude of the complex shear modulus ($|G^{*} |$), was calculated from the frequency-resolved data and indicates how much force is needed to deform the sample, i.e. how stiff or soft the FMs behave. At a frequency of 1 kHz, it shows a significant difference between the two tissue types. SD FMs are softer and thus easier to deform than CS FMs (1.9 kPa (IQR 1.6–2.4) vs. 4.7 kPa (IQR 3.8–5.6), *p* < 0.001). Samples were also measured using rotational shear rheology between two plates (see Fig. 2b). Plate rheology, the gold standard in material science, also quantifies the bulk, but depends more on the sample surface since a good contact between tissue and plates is required. No significant differences were found between 9 SD FMs and 16 CS FMs at a shear frequency of 1 Hz (0.56 kPa (IQR 0.47–1.09) vs. 0.67 kPa (IQR 0.48–1.19), *p* = 0.76). For enhanced spatial resolution, the outer surfaces of amnion and chorion, located on opposite sides of the FMs, were separately probed at multiple frequencies with atomic force microscopy (AFM), without prior detachment of the layers. They exhibit a relatively similar viscoelastic resistance ($|G^{*} |$) at a frequency of 100 Hz (see Fig. 2c) and a comparison between SD and CS FMs shows no significant differences (amnion: 0.91 kPa (IQR 0.26–1.73) vs. 0.72 kPa (IQR 0.49–0.95), *p* = 0.44; chorion: 0.67 kPa (IQR 0.6–0.9) vs. 0.6 kPa (IQR 0.41–0.92), *p* = 0.54). A similar behavior was found for the Young’s modulus, which is the typical elastic quantity obtained from AFM experiments (see Supplementary Fig. S1, amnion: 216 Pa (IQR: 71–335) vs. 124 Pa (IQR: 72–251), *p* = 0.73; chorion: 173 Pa (IQR 125–311) vs. 152 Pa (IQR 103–347), *p* = 0.90). Apart from the stiffness-based measurements, the loss factor can be calculated according to Eq. (5) (see Materials and Methods—Tabletop magnetic resonance elastography). Here, the ratio of the loss modulus to the storage modulus indicates the proportion of energy dissipated as heat (loss modulus) to the energy stored elastically (storage modulus) during a mechanical deformation process, reflecting how solid-like or fluid-like the tissue behaves. The median loss factor seems to be slightly higher in SD FMs compared to CS FMs in MRE measurements (see Fig. 2d, 0.95 (IQR 0.64–0.99) vs. 0.82 (IQR 0.63–0.89), *p* = 0.37). However, this difference is not significant and does not apply to rheometer measurements (see Fig. 2e, 0.2 (IQR 0.16–0.23) vs. 0.21 (IQR 0.17–0.23), *p* = 0.80) or AFM measurements (see Fig. 2f, amnion: 0.73 (IQR 0.53–0.81) vs. 0.81 (IQR 0.66–1.09), *p* = 0.20; chorion: 0.75 (IQR 0.68–0.92) vs. 0.7 (IQR 0.57–0.97), *p* = 0.44).

The mechanical resistance measured by the MRE was matched with the duration of pregnancy in a scatter plot (see Fig. 2g), to check for reduced mechanical stability in the FMs as pregnancy reaches full term. Linear trend lines are displayed, starting at the end of the 39th week of pregnancy to be consistent with the triphasic curve reported by Pressman et al.^31^. They indicate a slight increase in mechanical resistance for both groups, but the data are not significant (SD: R^2^ = 0.024, CS: R^2^ = 0.055). No significant trends were obtained for the rheometer measurements, shown in Fig. 2h (SD: R^2^ = 0.038, CS: R^2^ = 0.003). Due to the limited amount of data and the lack of significance, no fitting was performed. The mechanical dependency on the duration of pregnancy was also analyzed for all other physical parameters. No clear trends were found.

Besides MRE, the tabletop magnetic resonance setup can also be used to perform diffusion-weighted imaging (DWI) with b-values comparable to those typically used in clinical magnetic resonance imaging (MRI) setups (see Materials and Methods—Tabletop magnetic resonance elastography). The apparent diffusion coefficient (ADC), an indicator of water diffusion, is visualized in Fig. 2i. A lower ADC is typically observed in denser tissue structures^35^, while a more loosely organized ECM shows higher values. A marginally significant difference was found between 14 SD FMs and 12 CS FMs (1.78 × 10^−3^ mm^2^/s (IQR: 1.65–1.84 × 10^−3^) vs. 1.66 × 10^−3^ mm^2^/s (IQR: 1.60–1.73 × 10^−3^), *p* = 0.047). The SD FMs show an increased water diffusion. Thus, the water inside these FMs can move more freely compared to the CS FMs.

### FMs from spontaneous deliveries show a loose and more random fiber architecture in the ICT layer

**Fig. 3:**
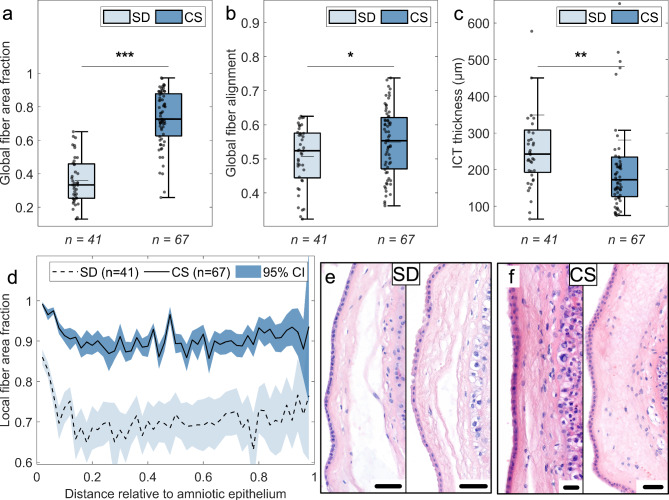
Analysis of the intermediate connective tissue (ICT) layer in histological images for fetal membranes (FMs) collected from spontaneous vaginal deliveries (SD) and primary cesarean sections (CS). (**a**–**c**) Global fiber area fraction (**a**, measured as the ratio of fiber area to total ICT area), global fiber alignment (**b**, measured by the averaged two-dimensional local nematic order parameter which can indicate random (0) or parallel (1) fiber arrangement), and ICT thickness (**c**, measured using the averaged spatial correlation length) were calculated for each image section and compared in boxplots. SD FMs show a significantly reduced global fiber area fraction (**a**, 0.33 (0.25–0.46) vs. 0.73 (0.63–0.88), *p* < 0.001), a more random fiber alignment (**b**, 0.52 (0.44–0.58) vs. 0.55 (0.47–0.62), *p* = 0.04), and a greater ICT thickness (**c**, 242 µm (192–308) vs. 172 µm (126–234), *p* = 0.001) compared to CS FMs. Individual samples are shown as dots, median (mean) values are represented as thick (thin) horizontal lines (*n* = number of image sections). Statistical significance was determined using a Wilcoxon rank-sum test: *p < 0.05, **p < 0.01, ***p < 0.001. (**d**) Local fiber area fraction as a function of the distance from the amniotic epithelium, shown for SD (dashed line, light blue confidence interval) and CS (solid line, dark blue confidence interval) FMs. Local properties were calculated per pixel, compared to a local circular neighborhood with a 5 µm radius, and averaged per distance for each image section. Distance-dependent results from all image sections were then averaged and displayed with the 95% confidence interval. SD FMs show a smaller local fiber area fraction compared to CS FMs with a pronounced decrease near the amniotic epithelium. (**e**–**f**) Representative histological image sections obtained from high resolution histological images of SD (**e**) and CS (**f**) FMs. The scale bars refer to a distance of 50 µm.

Digital cross sections through the FMs were extracted from high-resolution histological images and analyzed for ECM fiber structure using the eosin signal from H&E stains. Examples are shown in Fig. 3. The total fiber area fraction, measured as the ratio of the area occupied by eosin-stained ECM fibers to the total ICT area, shows significantly smaller values in SD FMs compared to CS FMs (see Fig. 3a, 0.33 (IQR 0.25–0.46) vs. 0.73 (IQR 0.63–0.88), *p* < 0.001). This area fraction is a measure of the total fiber density in the ICT layer. The local fiber area fraction (see Fig. 3d) gives insights into the distribution of fibers in the sample cross section. A reduced amount of fibers can be found towards the middle of the ICT area in both sample groups, but the SD FMs show a greater drop from the amniotic epithelium as well as smaller absolute values. As a second parameter to describe the ECM structure, fiber alignment was used, as denser fibers tend to align. Alignment has been quantified by the local nematic order parameter in the images. A value close to zero represents an isotropic fiber network, while a value of one represents a perfect parallel fiber alignment. The total fiber alignment, shown in Fig. 3b, is significantly smaller in SD FMs compared to the CS FMs (0.52 (IQR 0.44–0.58) vs. 0.55 (IQR 0.47–0.62), *p* = 0.04). The local fiber alignment shows a continuous decrease for both sample types with increasing distance to the epithelial cell layer of the amnion (see Supplementary Fig. S2). The average ICT layer thickness was measured for each cross-section as displayed in Fig. 3c. The SD FMs exhibit a clearly larger thickness compared to the CS FMs (242 µm (IQR 192–308) vs. 172 µm (IQR 126–234), *p* = 0.001). Note that the histological images of SD FMs show an overall lower eosin signal intensity in the ICT layer and a more porous structure compared to images of CS FMs (compare Fig. 3e–f). The increased ECM area fraction, ECM alignment, and eosin intensity in CS FMs are due to a higher ECM fiber density.

## Discussion

Although FMs are predominantly passive structures, they play a crucial role in the dynamics of the birth process^30,40–42^. Understanding the processes that initiate rupture of FMs enables better assessment of preterm birth risk and the development of new therapeutic strategies. Several research groups have used different methods to study the mechanical properties of FMs in order to explain their term and preterm rupture^18,23,30,40,41,43–47^. The stability of the membranes towards the end of pregnancy is decisive for whether the amniotic sac holds or ruptures. Our comprehensive biophysical approach combines methods for different size and time scales to investigate the mechanical footprint and structural changes of FMs.

Our MRE measurements reveal that FMs from vaginal deliveries are softer and thus weaker than FMs from cesarean sections (see Fig. 2a). This difference is not observed in rheometer and AFM measurements (see Fig. 2b–c). We attribute this discrepancy to the different working principles of these methods. The AFM only probes the outermost tissue layers by local compressive deformation, which is dominated by the epithelial cells of the amnion and the trophoblast cells of the chorion as well as their underlying basement membranes. FMs from spontaneous deliveries and FMs from cesarean sections behave similarly, indicating that the type of delivery does not affect the mechanical properties of these cell layers. The rheometer can measure the entire sample volume as the layers are rotated against one another. Shearing tests the mechanical coupling of FMs to their direct surroundings, as well as the interaction between individual layers and the force required to delaminate them. No rupture events could be recorded, as the surface properties of the FMs limited the maximum shear strain achievable within our experimental setup. Figure 2b shows two prominent outliers in the rheometer data for spontaneously delivered FMs, which have a substantial influence considering our sample size. Unlike AFM and plate rheology, which are more sensitive to the surfaces of the FMs, the MRE penetrates all layers of the tissue and probes the bulk viscoelasticity. The data clearly show that the inner regions of FMs from spontaneous vaginal deliveries are softer than those from cesarean sections. We conclude that the mechanical softening observed in spontaneously delivered FMs results from layer-specific degeneration processes which mainly affect the ECM, i.e. the ICT. This confirms that the mechanical response of FMs is highly dominated by the inner layers that form the ICT. They make up a large part of the sample volume (compare Fig. 1) and mainly consist of collagen, which plays a central role in the viscoelastic properties of tissues^35^.

The reduced mechanical stability found in our MRE experiments is confirmed by the results of our histological analysis (see Fig. 3). FMs from spontaneous deliveries show a smaller fiber area fraction, as expected for a dissolving ICT layer. Their ECM fibers are less aligned, which is the signature of less dense and dissolving membranes. Spontaneously delivered FMs show a looser fiber structure with large holes (see Fig. 3e), resulting in a more swollen and thicker ICT layer. These spaces can fill with water, potentially increasing both energy dissipation and water diffusion. In our combined MRE and DWI experiments, we observed a modest increase in energy dissipation and a marginally significant increase in water diffusion in FMs from spontaneous deliveries (see Fig. 2d,i). As more energy is dissipated during deformation—typically due to friction—the tissues behave more fluid-like. This fluidization might be reflected in the slightly increased water diffusion that we observed in spontaneously delivered FMs. The water can move more freely through the looser ECM. As the membranes soften and disintegrate, the probability of a rupture increases, which is the crucial prerequisite for spontaneous delivery. This supports the idea of a weakened ICT layer towards the end of pregnancy. In fact, most histological images of spontaneously delivered FMs showed a lower eosin intensity (i.e. ECM fiber intensity) in the ICT area compared to the images of FMs delivered by cesarean section, matching with our observed reduced fiber density (compare Fig. 3e–f). On the contrary, FMs from cesarean sections have a more compact ICT layer with more parallel aligned collagen fibers. This directly influences their mechanical properties, leading to higher stability and strength.

Overall, our findings demonstrate that FMs undergo a mechanical softening process during birth. FMs from spontaneous vaginal deliveries are mechanically softer and thus weaker than control FMs and show enhanced water diffusion. Our study provides evidence that this softening process is layer-specific and affects only the collagen-rich sublayers of FMs, where fibers become less dense and more randomly aligned during birth. Our biomechanical observations for spontaneously delivered FMs correlate well with reports by Meinert et al.^48^, who described that amnion and chorion are starting to separate towards the end of pregnancy, reducing the biomechanical strength of the FMs. This process is critical to facilitate membrane rupture at term.

The presented methods rely on different measurement principles sensitive to different scales and the absolute values are not always directly comparable, yet we still obtain a consistent overall picture. Nevertheless, the influence of physiological labor and uterine contractions on the histological and biomechanical properties of FMs remains uncertain. Therefore, the differences observed in our study could be partly due to the intralabor and postlabor state. The influence of permanent stretch to which the membranes are exposed in vivo could not be investigated with our experimental setups and remains unclear. In addition, FMs may exhibit local differences in composition, structure and mechanical properties. This heterogeneity can have a direct impact on the results, as only relatively small pieces of tissue were taken for the measurements. The correct timing of the softening process is of utmost importance for pregnancy. Due to the nature of the provided samples, we were unable to investigate this. However, given the prominent mechanical differences that we observe in term FMs, further studies on preterm FMs (both from patients with PPROM and with preterm delivery due to other reasons) are of great interest to investigate the timescale and progression of the mechanical softening process during pregnancy.

Ultimately, our understanding may help to predict, manage or even prevent PPROM events. MRE, which can be combined with DWI, proves to be a valuable method for monitoring the progression of mechanical instability in FMs. However, applying MRE and DWI in vivo may be difficult as the fetal membranes are quite thin and under constant motion. Although we successfully used histopathology to validate and explain our mechanical findings, it is also not a viable alternative, as it requires invasive biopsies. New methods based on ultrasound, optical coherence tomography, or MRI (e.g. slip interface imaging^49^), that measure fluctuations of the FMs as an indicator of their rigidity or are sensitive to their connectivity, may be suitable for future studies.

## Materials and methods

### Collection and preparation of samples

During a 5-month trial period, 93 tissue samples from 53 different patients (22 spontaneous vaginal deliveries, 31 primary cesarean sections) were collected and mechanically analyzed. In addition, 108 individual image sections from 13 patients (5 spontaneous vaginal deliveries, 8 primary cesarean sections) were used for histological analysis. FMs from spontaneous deliveries that went through the natural birth process were compared to FMs from cesarean sections, which served as control samples. The study was approved by the local ethics committee (Approval No. 540/21-ek, Date: 2021-12-20). All experiments were performed in accordance with relevant guidelines and regulations. Table 1 provides a brief overview of the demographic data of the patient population measured by each experimental method. Additional clinical information can be found in Table S1 in the supplementary section.

**Table 1:** Overview of patients included in the study and their associated information.

	MRE/MRI	Rheometer	AFM	Histological analysis
Number of patients, n (%)	26 (100)	25 (100)	24 (100)	13 (100)
SD	14 (54)	9 (36)	10 (42)	5 (38)
CS	12 (46)	16 (64)	14 (58)	8 (62)
Gestational age, mean ± std (days)	275.1 ± 5.9	273.2 ± 6.2	274 ± 5.7	275.8 ± 5.3
SD	275.5 ± 6.6	276.3 ± 8.3	274.3 ± 6.8	279.6 ± 5.7
CS	274.6 ± 5.2	271.5 ± 4.1	273.7 ± 4.9	273 ± 3.1
Unknown, n (%)	1 (4)	0 (0)	1 (4)	1 (8)
Age (mother), mean ± std (years)	31.7 ± 4.6	31.7 ± 5.9	31.3 ± 5.3	31.3 ± 5.6
SD	31.8 ± 3.6	28.2 ± 5.2	31.7 ± 5.1	30.8 ± 6.1
CS	31.6 ± 5.6	33.7 ± 5.6	31 ± 5.7	31.7 ± 5.8
Unknown, n (%)	1 (4)	0 (0)	1 (4)	1 (8)
BMI (mother), mean ± std (kg/m^2^)	26.4 ± 6.2	27.1 ± 5.4	28.4 ± 7.5	27.9 ± 8.1
SD	26 ± 3.7	26.7 ± 5.3	25.8 ± 4.2	24.5 ± 5.1
CS	26.9 ± 8.4	27.3 ± 5.6	30.5 ± 9.1	30.3 ± 9.3
Unknown, n (%)	2 (8)	0 (0)	2 (8)	1 (8)
Sex (baby), n male (%)	13 (50)	14 (56)	12 (50)	6 (46)
SD	6 (43)	5 (56)	4 (40)	4 (80)
CS	7 (58)	9 (56)	8 (57)	2 (25)
Unknown, n (%)	1 (4)	0 (0)	1 (4)	1 (8)
Weight (baby), mean ± std (g)	3344 ± 350	3318 ± 347	3243 ± 300	3377 ± 433
SD	3383 ± 346	3432 ± 336	3246 ± 301	3306 ± 284
CS	3308 ± 365	3253 ± 347	3239 ± 313	3428 ± 531
Unknown, n (%)	3 (12)	0 (0)	4 (4)	1 (8)

The percentages were rounded to integer values and refer to the total number of patients examined by the respective method or the total number of patients in the respective SD and CS cohort. As the information was not fully available for all patients, the number of samples with unknown information is added below. Please note that the number of tissue samples is larger than the number of patients for the AFM method, as usually two pieces of tissue from the same patient were prepared and measured separately to assess the inner and outer layers of the FMs (see Materials and Methods—Atomic Force Microscopy). For each patient, several small image sections were selected from the original image and analyzed (see Materials and Methods—Histological Analysis). A mechanical characterization of the same patient with all three measurement methods could not always be realized, which is why the underlying patient populations strongly overlap but are not identical. In total, 93 tissue samples from 53 different patients were mechanically analyzed in this study. 108 individual image sections from 13 patients were used for the histological analysis. More detailed clinical information can be found in Table S1 in the supplementary section.

Human FMs were collected with informed consent immediately after placental delivery from pre-selected term pregnancies (> 38 + 0 weeks of gestation, vaginal delivery or primary cesarean section, no operatively assisted vaginal delivery, singleton, no administration of external prostaglandins or uterotonics, no bleeding or clinical signs of infection) by medical staff at the Department of Obstetrics of the Leipzig University Hospital. All vaginal deliveries had a spontaneous onset of labor without prior mechanical manipulation. All primary cesarean sections were performed with intact membranes prior to the onset of labor. Thus, the collected FMs were still intact and not weakened or damaged by uterine contractions or biochemical weakening processes. We can therefore assume that they represent the typical viscoelastic behavior of FMs before the onset of labor. The FMs used for mechanical measurements were stored refrigerated at 4 °C in a 0.9% isotonic saline solution (B. Braun, Melsungen, Germany) directly after delivery until they were collected, prepared and measured in less than 12 h at the Peter Debye Institute for Soft Matter Physics in Leipzig. FMs used for histological imaging were fixed in a 4% formaldehyde solution (Otto Fischar GmbH & Co. KG, Saarbrücken, Germany) right after delivery and refrigerated for a few days. They were then collectively sent to the Institute for Histology, Cytology and Molecular Diagnostics at the Pathology Hamburg-West.

### Tabletop magnetic resonance elastography

**Fig. 4:**
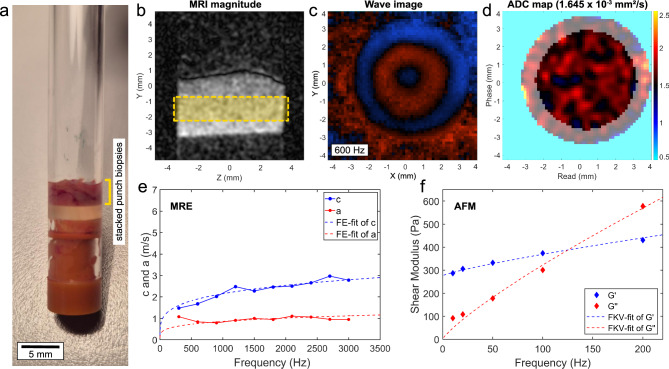
Overview of the mechanical testing and analysis of samples with MRE/MRI and AFM. (**a**) Image of a prepared sample for the tabletop magnetic resonance setup. Several punch biopsies were stacked on top of each other (see yellow marking) to increase the sample volume. (**b**) Vibration amplitude image of the sample in the glass tube. The yellow rectangle shows the typical area in which the signals were recorded. The slice was chosen with a thickness of 1.5 mm to exclude any surface effects. (**c**) Typical wave image recorded at a frequency of 600 Hz showing the propagation of the mechanical shear wave through the sample volume. The colors indicate a positive (red) or negative (blue) displacement along the z-axis. (**d**) Typical ADC map showing the average water diffusion for each pixel (averaged over the entire z range). The light grey area was excluded from further analysis as it often contained water inclusions located at the edge between the sample and the glass wall (indicated by the high diffusion coefficient, shown in yellow). (**e**) Shear wave speed $c$ (blue) and shear wave penetration rate $a$ (red), calculated from the wave images, shown for a representative example. Both properties were fitted using a fractional element model (dashed lines). (**f**) Shear storage (blue) and shear loss modulus (red) obtained from AFM measurements, shown for a representative example. The fractional Kelvin–Voigt model fits (dashed lines) show a crossover which was typically observed in the measured frequency range.

During sample preparation, several 8 mm punch biopsies were taken from the FMs while they were kept in a cooled state. To achieve the necessary sample volume, at least three punch biopsies were stacked on top of each other in a sandwich-like manner. They were then positioned at the bottom of a 7 mm diameter glass tube, placed above a silicon shock absorber (see Fig. 4a). A small cotton wool ball soaked in phosphate buffered saline (PBS, Thermo Fisher Scientific, Waltham, USA) was added on top of the tube to maintain nearly 100% humidity and prevent the sample from drying out during the measurement. The tube was then sealed at the top and bottom with small plastic plugs.

Measurements were performed at 37 °C using a tabletop magnetic resonance imaging (MRI) scanner (Pure Devices, Würzburg, Germany) equipped with a 0.5 T permanent magnet modified by a DC 600 gradient amplifier (Pure Devices, Würzburg, Germany). A piezoelectric driver (Piezosystem Jena, Jena, Germany) allows the coverage of frequencies from 200 up to 6000 Hz. The sample tube was connected to the piezoelectric driver from above such that the punch biopsies were positioned in the center of the 10 mm bore of the MRI scanner. The vibrations of the driver were coupled into the sample via the glass walls of the tube, generating shear waves in the sample volume (see Fig. 4b–c). The FMs were suitable for viscoelastic measurements between 300 and 3000 Hz. With an interval of 300 Hz this resulted in 10 assessed frequencies.

The acquired data were then post-processed as described by Braun et al.^50^. In brief, custom MATLAB scripts (MathWorks, Natick, USA) were used to unwrap the acquired phase data and perform a Fourier transformation in time to extract complex wave images. These images were then used to derive the complex wavenumber $k^{*} = k^{\prime } + ik^{\prime \prime }$ by applying analytical solutions for shear waves in a z-infinite cylinder^51,52^. From that, the shear wave speed $c$ and the shear wave penetration rate $a$ could be calculated for each frequency $f$:

1 $$c = \frac{2\pi f}{{k^{\prime } }}\;{\text{and}}\;a = \frac{f}{{k^{\prime \prime } }}$$

Under the assumption that there are no compressional waves created in the sample volume, the complex shear wave velocity is given by

2 $$c^{*} \left( \omega \right) = \sqrt {\frac{{G^{*} \left( \omega \right)}}{\rho }}$$

where $\rho$ represents the materials density. Using a plane wave model, $c$ and $a$ could then be expressed in terms of the frequency-dependent complex shear modulus $G^{*} \left( \omega \right)$:

3 $$c\left( \omega \right) = \sqrt {\frac{{2\left| {G^{*} \left( \omega \right)} \right|}}{{\rho \left( {1 + cos\left( \varphi \right)} \right)}}} \;{\text{and}}\;a\left( \omega \right) = \frac{1}{2\pi } \sqrt {\frac{{2\left| {G^{*} \left( \omega \right)} \right|}}{{\rho \left( {1 - cos\left( \varphi \right)} \right)}}}$$

To derive the complex shear modulus for an arbitrary frequency, a viscoelastic fractional element model that is based on a generalized fractal Maxwell model^53^ was used (example fit curves are shown in Fig. 4e)

4 $$G\left( f \right)^{*} = \mu^{1 - \alpha } \eta^{\alpha } \left( {i2\pi f} \right)^{\alpha } = G^{\prime } \left( f \right) + iG^{\prime \prime } \left( f \right)$$

where $\mu$ can be interpreted as a stiffness parameter, $\alpha$ can be related to the loss angle by multiplication with $\pi /2$, and $\eta$ represents the viscosity (typically set to 1 to obtain a two-parameter model). The model has shown good applicability on both eukaryotic cells^54^ and biological tissues^53^. The real and imaginary parts of the complex shear modulus represent the shear storage modulus ($G^{\prime }$) and the shear loss modulus ($G^{\prime \prime }$), which represent the amount of energy temporarily stored or lost during the deformation process. The loss factor was then calculated as the ratio of shear loss modulus to shear storage modulus via

5 $$tan\left( \delta \right) = \frac{{G^{\prime \prime } }}{{G^{\prime } }}$$

and it indicates how much energy dissipated during the process (i.e. how solid- or fluid-like the sample behaves). For MRE measurements a frequency of 1 kHz was chosen to represent the magnitude of the complex shear modulus $\left| {G^{*} } \right|$ in this study.

In addition, diffusion-weighted imaging (DWI) was performed on each sample using the tabletop MRI scanner, maintaining the same conditions (temperature, measurement volume) as those used for MRE measurements. The DWI involved applying five different magnetic diffusion gradient strengths (“b-values”), ranging from 50 to 750 s/mm^2^ in increments of 175 s/mm^2^, a range that is typically used in the clinic. The apparent diffusion coefficient (ADC) was then calculated from the signal intensity decay associated with increasing b-values and averaged over the entire sample volume. To avoid falsification of the results by water inclusions, the outer edge area was excluded from the analysis (as shown in Fig. 4d).

### Rheometer

Cooled FMs were cut into 2 × 2 cm^2^ pieces in a Petri dish using a scalpel and subsequently stored in a tube with modified ringer solution (B. Braun, Melsungen, Germany) to nourish the tissue. The prepared samples were then measured with a TA ARES rheometer (Waters corporation, Milford, USA) to probe their viscoelastic response under rotational shear.

For rheological measurements, an 8 mm parallel plate setup was used and maintained at 20 °C during the whole measurement. Sandpaper (grit size 80) was attached to the metallic bottom plate with double-sided adhesive tape. The samples were then clamped with a constant mean contact force of 0.22 N and the gap width was adjusted accordingly. Frequency sweeps were performed at 1% strain, ranging from 10^−2^ to 10 Hz.

The measured data were exported and analyzed with the device-specific software TA orchestrator V7.2.0.2 (TA instruments, New Castle, USA) and returned as components of the complex shear modulus ($G^{*}$) for the entire frequency range according to the right-hand side of Eq. (4). The loss factor was calculated according to Eq. (5). All data were then analyzed with self-written MATLAB scripts. For comparison with the other experimental methods in this study, it was decided to present the data in the middle of the covered frequency range, at 1 Hz.

### Atomic force microscopy

After the cooled samples arrived at the laboratory, two small pieces of approximately 3 × 3 mm^2^ were carefully cut out with a scalpel and glued on a thin glass slide using Histoacryl tissue adhesive (B. Braun Surgical, Rubí, Spain). The tissues were arranged so that one sample was placed with the amnion and the other with the chorion facing upwards. This allowed a separate non-destructive measurement of the surfaces of amnion and chorion (i.e. the amniotic epithelium and the trophoblast, compare Fig. 1). The glass slide was glued in a 40 mm petri dish and submerged in 2–3 ml PBS solution.

Measurements were carried out at room temperature right after preparation using a Zeiss upright AxioZoom.V16 fluorescence microscope (Carl Zeiss Microscopy GmbH, Jena, Germany) in combination with the NanoWizard 4 XP setup and a 300 µm HybridStage (JPK BioAFM Business, Berlin, Germany). NanoWorld Pointprobe CONT cantilevers (NanoWorld AG, Neuchâtel, Switzerland) were modified with small polystyrene beads (6 µm diameter) to increase the contact area between sample and cantilever during the indentation process. At least 1600 individual force indentation curves were recorded for each sample surface with a set force of 2 nN, covering an area of about 600 × 600 µm^2^. Between each approach and retraction phase, the cantilever was paused and held at constant height as soon as it reached the set force and the piezo actuator performed sinusoidal oscillations in the range of 10–200 Hz, which caused the cantilever to vibrate. This allowed a measurement of the elastic and viscoelastic sample properties within the same experiment.

The recorded force-indentation data was pre-processed with the JPK data processing software V7.1.18 (Bruker Nano GmbH, Berlin, Germany) and the Young’s modulus was derived from a Hertz model fit^55,56^. Non-elastic properties were analyzed with the built in JPK microrheology module. The vibration data were fitted with a Sneddon model^56^ for spherically shaped indenters, which allowed the calculation of shear storage and shear loss moduli. All data were subsequently post-processed using self-written MATLAB scripts. Therefore, the viscoelastic information was fitted over the whole frequency range by a fractional derivative Kelvin–Voigt model^57–59^

6 $$G\left( f \right)^{*} = \mu^{1 - \alpha } \eta^{\alpha } \left( {i2\pi f} \right)^{\alpha } + \mu_{0}$$

which is an extended version of the previously mentioned FE model (compare Eq. 4). It introduces the stiffness-parameter $\mu_{0}$, which allows a crossover of storage and loss moduli as typically observed in the AFM data in the measured frequency range (representative curves are shown in Fig. 4f). Finally, the obtained viscoelastic data were evaluated at a frequency of 100 Hz.

### Histological analysis

After arrival at the Pathology Hamburg-West, the formalin fixed FMs were sectioned and embedded in cassettes, followed by a standard tissue preparation protocol for paraffin embedding and slicing. Afterwards, they were stained using a standard H&E-staining procedure. Each slide was optically inspected and digitized using the Pannoramic 1000 Flash IV Scanner of 3DHISTECH with a Zeiss Apochromat 40 × objective (Carl Zeiss Microscopy GmbH, Jena, Germany). The high-resolution histological images were further analyzed at the Peter Debye Institute for Soft Matter Physics in Leipzig with computational image analysis.

All image sections were processed and analyzed using self-written MATLAB scripts. First, the raw histological images were visually examined for suitable subsections in which the ICT areas could be clearly identified. The ICT area was then annotated by hand in all image sections (ICT mask). During image analysis, the eosin signal representing the ECM fibers was extracted from the RGB images using a custom color deconvolution code based on the work of Macenko et al.^60^. A logical mask was determined based on Otsu’s method^61^ and generated for each image section (eosin mask). The fiber area fraction was then calculated as the ratio of the eosin area to the overall ICT area. In addition, the ICT masks were used to estimate the thickness of the ICT layer by calculating their radially averaged spatial correlation length via the Wiener-Khinchin theorem. The eosin intensities were additionally used to calculate the fiber alignment. Therefore, the orientations of the eosin signal were estimated as the eigenvectors corresponding to the lowest eigenvalue of the structure tensor (window width 1 µm) at each pixel. From the resulting orientation field, the two-dimensional scalar nematic order parameter was calculated as

7 $$S = \left\langle {cos\left( {2\phi } \right)} \right\rangle$$

here $\phi$ represents the angle between the local pixel orientation and the pixels in its local environment, which was chosen as a circular area with a radius of 5 µm.

### Statistical analysis

Statistical analysis and visualization of the processed data was performed with MATLAB. First, the Shapiro–Wilk test^62^ was applied to assess the normality of each group, as it is suitable for small datasets. Since the assumption of normality was not met for all groups, the two-sided Wilcoxon rank-sum test^63^ was used to compare two groups at a time and assess statistical significance. Statistical information is presented in each figure, with asterisks indicating the level of significance (*p < 0.05, **p < 0.01, ***p < 0.001). In the text, statistical data are reported as (median 1 (interquartile range 1) vs. median 2 (interquartile range 2), *p* value).

## Supplementary Information

Supplementary Information.

## Data Availability

The datasets generated during and/or analyzed during the current study are available in the “fetal-membranes-project” repository: https://github.com/PhilipFriedrich/fetal-membranes-project.git.

## References

[1] Truong, N., Menon, R. & Richardson, L. The role of fetal membranes during gestation, at term, and preterm labor. *Placenta Reprod. Med.* **2**, 4 (2023). 38304894 10.54844/prm.2022.0296 PMC10831903

[2] Zhu, C. et al. Nutritional and physiological regulation of water transport in the conceptus. *Adv. Exp. Med. Biol.* **1354**, 109–125 (2022). 34807439 10.1007/978-3-030-85686-1_6

[3] Moore, R. M., Mansour, J. M., Redline, R. W., Mercer, B. M. & Moore, J. J. The physiology of fetal membrane rupture: Insight gained from the determination of physical properties. *Placenta* **27**, 1037–1051 (2006). 16516962 10.1016/j.placenta.2006.01.002

[4] Friel, N. A. et al. Amniotic fluid, cells, and membrane application. *Oper. Tech. Sports Med.* **25**, 20–24 (2017).

[5] Mercer, B. M. Preterm premature rupture of the membranes. *Obstet. Gynecol.* **101**, 178–193 (2003). 12517665 10.1016/s0029-7844(02)02366-9

[6] Chawanpaiboon, S. et al. Global, regional, and national estimates of levels of preterm birth in 2014: A systematic review and modelling analysis. *Lancet Glob. Health* **7**, e37–e46 (2019). 30389451 10.1016/S2214-109X(18)30451-0 PMC6293055

[7] Walani, S. R. Global burden of preterm birth. *Int. J. Gynecol. Obstet.* **150**, 31–33 (2020). 10.1002/ijgo.13195 32524596

[8] Naumann, J., Koppe, N., Thome, U. H., Laube, M. & Zink, M. Mechanical properties of the premature lung: From tissue deformation under load to mechanosensitivity of alveolar cells. *Front. Bioeng. Biotechnol.*10.3389/fbioe.2022.964318 (2022). 36185437 10.3389/fbioe.2022.964318 PMC9523442

[9] Ward, R. M. & Beachy, J. C. Neonatal complications following preterm birth. *BJOG Int. J. Obstet. Gynaecol.* **110**, 8–16 (2003). 10.1016/s1470-0328(03)00012-0 12763105

[10] Hislop, A. A., Wigglesworth, J. S., Desai, R. & Aber, V. The effects of preterm delivery and mechanical ventilation on human lung growth. *Early Hum. Dev.* **15**, 147–164 (1987). 3608888 10.1016/0378-3782(87)90003-x

[11] Mitrogiannis, I. et al. Risk factors for preterm birth: An umbrella review of meta-analyses of observational studies. *BMC Med.* **21**, 494 (2023). 38093369 10.1186/s12916-023-03171-4 PMC10720103

[12] Cobo, T., Kacerovsky, M. & Jacobsson, B. Risk factors for spontaneous preterm delivery. *Int. J. Gynecol. Obstet.* **150**, 17–23 (2020). 10.1002/ijgo.13184 32524595

[13] Wl, L., Wh, C. & Ph, W. Risk factors associated with preterm premature rupture of membranes (PPROM). *Taiwan. J. Obstet. Gynecol.* **60**, 805–806 (2021). 34507652 10.1016/j.tjog.2021.07.004

[14] Dayal, S. & Hong, P. L. Premature rupture of membranes. in *StatPearls* (StatPearls Publishing, Treasure Island (FL), 2024). 30422483

[15] Samejima, T., Yamashita, T., Takeda, Y. & Adachi, T. Identifying the associated factors with onset of preterm PROM compared with term PROM: A retrospective cross-sectional study. *Taiwan. J. Obstet. Gynecol.* **60**, 653–657 (2021). 34247802 10.1016/j.tjog.2021.05.012

[16] Romero, R., Dey, S. K. & Fisher, S. J. Preterm labor: One syndrome, many causes. *Science* **345**, 760–765 (2014). 25124429 10.1126/science.1251816 PMC4191866

[17] Romero, R. et al. Vaginal progesterone in women with an asymptomatic sonographic short cervix in the midtrimester decreases preterm delivery and neonatal morbidity: A systematic review and metaanalysis of individual patient data. *Am. J. Obstet. Gynecol.* **206**(124), e1-19 (2012). 10.1016/j.ajog.2011.12.003 PMC3437773 22284156

[18] Shilo, D. & Shalev, E. New insights on the biomechanics of the fetal membrane. *Front. Biosci. Sch. Ed.* **15**, 6 (2023). 10.31083/j.fbs1502006 37401507

[19] Oyen, M. L. et al. Uniaxial and biaxial mechanical behavior of human amnion. *J. Mater. Res.* **20**, 2902–2909 (2005).

[20] Leal-Marin, S. et al. Human amniotic membrane: A review on tissue engineering, application, and storage. *J. Biomed. Mater. Res. B Appl. Biomater.* **109**, 1198–1215 (2021). 33319484 10.1002/jbm.b.34782

[21] Jirsova, K. & Jones, G. L. A. Amniotic membrane in ophthalmology: properties, preparation, storage and indications for grafting—a review. *Cell Tissue Bank.* **18**, 193–204 (2017). 28255771 10.1007/s10561-017-9618-5

[22] Eichholz, H. M. et al. Anatomy of the fetal membranes: Insights from spinning disk confocal microscopy. *Arch. Gynecol. Obstet.* **309**, 1919–1923 (2024). 37184578 10.1007/s00404-023-07070-0 PMC11018647

[23] Oyen, M. L., Cook, R. F. & Calvin, S. E. Mechanical failure of human fetal membrane tissues. *J. Mater. Sci. Mater. Med.* **15**, 651–658 (2004). 15346731 10.1023/b:jmsm.0000030205.62668.90

[24] Calvin, S. E. & Oyen, M. L. Microstructure and mechanics of the chorioamnion membrane with an emphasis on fracture properties. *Ann. N. Y. Acad. Sci.* **1101**, 166–185 (2007). 17332077 10.1196/annals.1389.009

[25] Joyce, E. M., Moore, J. J. & Sacks, M. S. Biomechanics of the fetal membrane prior to mechanical failure: Review and implications. *Eur. J. Obstet. Gynecol. Reprod. Biol.* **144**, S121–S127 (2009). 19303191 10.1016/j.ejogrb.2009.02.014 PMC2688645

[26] Marom, Y., Gengrinovitch, S., Shalev, E. & Shilo, D. Collagen bundling and alignment in equibiaxially stretched human amnion. *J. Biomech.* **108**, 109896 (2020). 32636005 10.1016/j.jbiomech.2020.109896

[27] Strauss, J. F. Extracellular matrix dynamics and fetal membrane rupture. *Reprod. Sci. Thousand Oaks Calif* **20**, 140–153 (2013). 10.1177/1933719111424454 PMC3826277 22267536

[28] Uchide, K. et al. Matrix metalloproteinase-9 and tensile strength of fetal membranes in uncomplicated labor. *Obstet. Gynecol.* **95**, 851–855 (2000). 10831980 10.1016/s0029-7844(00)00811-5

[29] Goldman, S., Weiss, A., Eyali, V. & Shalev, E. Differential activity of the gelatinases (matrix metalloproteinases 2 and 9) in the fetal membranes and decidua, associated with labour. *Mol. Hum. Reprod.* **9**, 367–373 (2003). 12771238 10.1093/molehr/gag040

[30] Kumar, D. et al. Proinflammatory cytokines found in amniotic fluid induce collagen remodeling, apoptosis, and biophysical weakening of cultured human fetal membranes. *Biol. Reprod.* **74**, 29–34 (2006). 16148217 10.1095/biolreprod.105.045328

[31] Pressman, E. K., Cavanaugh, J. L. & Woods, J. R. Physical properties of the chorioamnion throughout gestation. *Am. J. Obstet. Gynecol.* **187**, 672–675 (2002). 12237646 10.1067/mob.2002.125742

[32] Buerzle, W. et al. Multiaxial mechanical behavior of human fetal membranes and its relationship to microstructure. *Biomech. Model. Mechanobiol.* **12**, 747–762 (2013). 22972367 10.1007/s10237-012-0438-z

[33] Xie, X. et al. Effect of non-linear strain stiffening in eDAH and unjamming. *Soft Matter* **20**, 1996–2007 (2024). 38323652 10.1039/d3sm00630a PMC10900305

[34] Warmt, E. et al. Differences in cortical contractile properties between healthy epithelial and cancerous mesenchymal breast cells. *New J. Phys.* **23**, 103020 (2021).

[35] Sauer, F. et al. Collagen networks determine viscoelastic properties of connective tissues yet do not hinder diffusion of the aqueous solvent. *Soft Matter* **15**, 3055–3064 (2019). 30912548 10.1039/c8sm02264j

[36] Sauer, F. et al. Changes in tissue fluidity predict tumor aggressiveness in vivo. *Adv. Sci.* **10**(26), 2303523 (2023). 10.1002/advs.202303523 PMC10502644 37553780

[37] Fuhs, T. et al. Rigid tumours contain soft cancer cells. *Nat. Phys.* **18**, 1510–1519 (2022).

[38] Fuhs, T. et al. Combining atomic force microscopy and fluorescence-based techniques to explore mechanical properties of naive and ischemia-affected brain regions in mice. *Sci. Rep.* **13**, 12774 (2023). 37550347 10.1038/s41598-023-39277-1 PMC10406906

[39] Sauer, F. et al. Whole tissue and single cell mechanics are correlated in human brain tumors. *Soft Matter* **17**, 10744–10752 (2021). 34787626 10.1039/d1sm01291f PMC9386686

[40] Menon, R., Richardson, L. S. & Lappas, M. Fetal membrane architecture, aging and inflammation in pregnancy and parturition. *Placenta* **79**, 40–45 (2019). 30454905 10.1016/j.placenta.2018.11.003 PMC7041999

[41] Menon, R. Fetal inflammatory response at the fetomaternal interface: A requirement for labor at term and preterm. *Immunol. Rev.* **308**, 149–167 (2022). 35285967 10.1111/imr.13075 PMC9188997

[42] Menon, R. Human fetal membranes at term: Dead tissue or signalers of parturition?. *Placenta* **44**, 1–5 (2016). 27452431 10.1016/j.placenta.2016.05.013 PMC5375105

[43] Verbruggen, S. W., Oyen, M. L., Phillips, A. T. M. & Nowlan, N. C. Function and failure of the fetal membrane: Modelling the mechanics of the chorion and amnion. *PLoS ONE* **12**, e0171588 (2017). 28350838 10.1371/journal.pone.0171588 PMC5370055

[44] Skinner, S. J., Campos, G. A. & Liggins, G. C. Collagen content of human amniotic membranes: Effect of gestation length and premature rupture. *Obstet. Gynecol.* **57**, 487–489 (1981). 7243099

[45] Bhunia, S. et al. New approaches suggest term and preterm human fetal membranes may have distinct biomechanical properties. *Sci. Rep.* **12**, 5109 (2022). 35332209 10.1038/s41598-022-09005-2 PMC8948223

[46] Helmig, R., Oxlund, H., Petersen, L. K. & Uldbjerg, N. Different biomechanical properties of human fetal membranes obtained before and after delivery. *Eur. J. Obstet. Gynecol. Reprod. Biol.* **48**, 183–189 (1993). 8335136 10.1016/0028-2243(93)90086-r

[47] Arikat, S. et al. Separation of amnion from choriodecidua is an integral event to the rupture of normal term fetal membranes and constitutes a significant component of the work required. *Am. J. Obstet. Gynecol.* **194**, 211–217 (2006). 16389034 10.1016/j.ajog.2005.06.083

[48] Meinert, M. et al. Labour induces increased concentrations of biglycan and hyaluronan in human fetal membranes. *Placenta* **28**, 482–486 (2007). 17125833 10.1016/j.placenta.2006.09.006

[49] Zheng, K. et al. Improved quantification of tumor adhesion in meningiomas using MR elastography-based slip interface imaging. *PLoS ONE* **19**, e0305247 (2024). 38917107 10.1371/journal.pone.0305247 PMC11198761

[50] Braun, J. et al. A compact 0.5 T MR elastography device and its application for studying viscoelasticity changes in biological tissues during progressive formalin fixation. *Magn. Reson. Med.* **79**, 470–478 (2018). 28321914 10.1002/mrm.26659

[51] Okamoto, R. J., Clayton, E. H. & Bayly, P. V. Viscoelastic properties of soft gels: comparison of magnetic resonance elastography and dynamic shear testing in the shear wave regime. *Phys. Med. Biol.* **56**, 6379 (2011). 21908903 10.1088/0031-9155/56/19/014 PMC3178746

[52] Yasar, T. K., Royston, T. J. & Magin, R. L. Wideband MR elastography for viscoelasticity model identification. *Magn. Reson. Med.* **70**, 479–489 (2013). 23001852 10.1002/mrm.24495 PMC3556381

[53] Klatt, D. et al. Viscoelastic properties of liver measured by oscillatory rheometry and multifrequency magnetic resonance elastography. *Biorheology* **47**, 133–141 (2010). 20683156 10.3233/BIR-2010-0565

[54] Djordjević, V. D., Jarić, J., Fabry, B., Fredberg, J. J. & Stamenović, D. Fractional derivatives embody essential features of cell rheological behavior. *Ann. Biomed. Eng.* **31**, 692–699 (2003). 12797619 10.1114/1.1574026

[55] Hertz, H. Über die berührung fester elastischer Körper. *J Reine Angew. Math.* **92**, 156 (1881).

[56] Sneddon, I. N. The relation between load and penetration in the axisymmetric boussinesq problem for a punch of arbitrary profile. *Int. J. Eng. Sci.* **3**, 47–57 (1965).

[57] Bonfanti, A., Kaplan, J. L., Charras, G. & Kabla, A. Fractional viscoelastic models for power-law materials. *Soft Matter* **16**, 6002–6020 (2020). 32638812 10.1039/d0sm00354a

[58] Jóźwiak, B., Orczykowska, M. & Dziubiński, M. Fractional generalizations of Maxwell and Kelvin–Voigt models for biopolymer characterization. *PLoS ONE* **10**, e0143090 (2015). 26599756 10.1371/journal.pone.0143090 PMC4658031

[59] Farno, E., Baudez, J.-C. & Eshtiaghi, N. Comparison between classical Kelvin–Voigt and fractional derivative Kelvin–Voigt models in prediction of linear viscoelastic behaviour of waste activated sludge. *Sci. Total Environ.* **613–614**, 1031–1036 (2018). 28950665 10.1016/j.scitotenv.2017.09.206

[60] Macenko, M. *et al.* A method for normalizing histology slides for quantitative analysis. in *2009 IEEE International Symposium on Biomedical Imaging: From Nano to Macro* 1107–1110 (2009). 10.1109/ISBI.2009.5193250.

[61] Otsu, N. A threshold selection method from gray-level histograms. *IEEE Trans. Syst. Man Cybern.* **9**, 62–66 (1979).

[62] Shapiro, S. S. & Wilk, M. B. An analysis of variance test for normality (complete samples). *Biometrika* **52**, 591–611 (1965).

[63] Wilcoxon, F. Individual comparisons by ranking methods. In *Breakthroughs in Statistics: Methodology and Distribution* (eds Kotz, S. & Johnson, N. L.) 196–202 (Springer, 1992). 10.1007/978-1-4612-4380-9_16.

[64] Roy, A. & Griffiths, S. Intermediate layer contribution in placental membrane allografts. *J. Tissue Eng. Regen. Med.* **14**, 1126–1135 (2020). 32592334 10.1002/term.3086

[65] Mamede, A. C. et al. Amniotic membrane: From structure and functions to clinical applications. *Cell Tissue Res.* **349**, 447–458 (2012). 22592624 10.1007/s00441-012-1424-6

[66] Sabol, T. J., Tran, G. S., Matuszewski, J. & Weston, W. W. Standardized reporting of amnion and amnion/chorion allograft data for wound care. *Health Sci. Rep.* **5**, e794 (2022). 36032519 10.1002/hsr2.794 PMC9399452

[67] Menon, R., Radnaa, E., Behnia, F. & Urrabaz-Garza, R. Isolation and characterization human chorion membrane trophoblast and mesenchymal cells. *Placenta* **101**, 139–146 (2020). 32979718 10.1016/j.placenta.2020.09.017 PMC7584780

