# Supplementary material for: Birth mode is associated with layer-specific mechanical changes in fetal membranes
Source: Sci Rep. 2025 Jun 20;15:20132. doi: 10.1038/s41598-025-04752-4 (PMC12181243; doi:10.1038/s41598-025-04752-4)
Supplement: Supplementary file 1 — Supplementary Information. [file 41598_2025_4752_MOESM1_ESM.pdf]

Supplementary Information

Main manuscript: "Birth mode is associated with layer-specific mechanical changes in fetal membranes"

Authors: Philip Friedrich [1 †], Hanna Grubitzsch [2 †], Benjamin Wolf [3], Hannah M. Eichholz [1,4,5], Cary Tutmarc [1], Pablo Gottheil [1], Frank Sauer [1,6], Alissa Cornelis [2], Anne-Sophie Wegscheider [7], Bahriye Aktas [3], Josef A. Käs [1‡], Holger Stepan [2‡*]

[1] Peter Debye Institute for Soft Matter Physics, Leipzig University, 04103 Leipzig, Germany

[2] Department of Obstetrics, University Hospital Leipzig, 04103 Leipzig, Germany

[3] Department of Gynecology, University Hospital Leipzig, 04103 Leipzig, Germany

[4] Leipzig Institute for Meteorology, Leipzig University, 04103 Leipzig, Germany

[5] Center for Scalable Data Analytics and Artificial Intelligence, Leipzig University, 04105 Leipzig, Germany

[6] Department of Radiology, Charité - Universitätsmedizin Berlin, 10117 Berlin, Germany

[7] MVZ Prof. Dr. med. A. Niendorf Pathologie Hamburg-West GmbH, Institute for Histology, Cytology and Molecular Diagnostics, 22767 Hamburg, Germany

[†] These authors contributed equally to this work

[‡] These authors share the last authorship

[*] Corresponding author: Holger Stepan (Holger.Stepan@medizin.uni-leipzig.de)

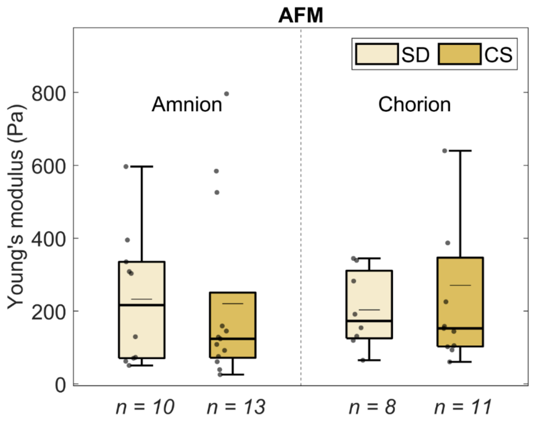

Supplementary Fig. S1: Surface elasticity of fetal membranes (FMs) collected from spontaneous vaginal deliveries (SD) and primary cesarean sections (CS) measured by AFM. Surfaces of amnion and chorion (i.e. the amniotic epithelium and the trophoblast) were measured separately. The elasticity was measured by the elastic modulus (i.e. "Young's modulus"). No significant differences were found between SD and CS FMs (amnion: 216 Pa (IQR: 71-335) vs. 124 Pa (IQR: 72-251), $p = .73$; chorion: 173 Pa (IQR: 125-311) vs. 152 Pa (IQR: 103-347), $p = .90$). Individual samples are shown as dots, median (mean) values are represented as thick (thin) horizontal lines (n = number of samples). Statistical significance was determined using a Wilcoxon rank-sum test: $^*p < .05$, $^{**}p < .01$, $^{***}p < .001$.

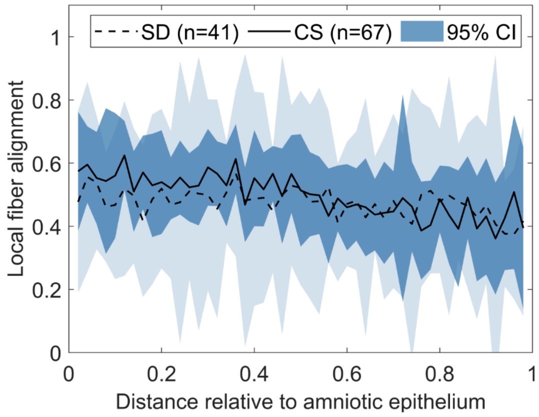

Supplementary Fig. S2: Local fiber alignment as a function of the distance from the amniotic epithelium for fetal membranes (FMs) collected from spontaneous vaginal deliveries (SD) and primary cesarean sections (CS). The fiber alignment is measured by the averaged two-dimensional local nematic order parameter which can indicate random (0) or parallel (1) fiber alignment. Local properties were calculated per pixel, compared to a local circular neighborhood with a 5 µm radius, and averaged per distance for each image section. Distance-dependent results from all image sections were then averaged and displayed with the 95 % confidence interval (*n* = number of image sections). SD (dashed line, light blue confidence interval) and CS (solid line, dark blue confidence interval) FMs were compared. Both delivery types show a slight decrease in fiber alignment with increasing distance to the amniotic epithelium.

FMs	Delivery	MRE	Rheo	AFM	Histo	Sex (m/f)	Height (cm)	Weight (g)	Head size (cm)	GA (week+day)	Parity	M age (y)	M height (cm)	M weight (kg)	M BMI (kg/m^2)	M nicotin (y/n)
1	SD	x				f	48	3010	35	38+6	II	29	164	73.00	27.10	y
2	SD	x		x		m	49	3330	36	39+1	II	37	169	65.80	23.00	n
3	CS	x		x		m	47.5	2700	32	38+0	II	19				
4	CS	x				m	47	3070	35	39+0	II	32	162	58.00	22.10	n
5	CS	x		x		f	50	3290	35	40+3	X	35	164	65.00	24.20	
6	CS	x		x		f	51	3610	35	39+1	I	26	179	69.00	21.50	
7	SD	x		x		f	48	2900	32	38+1	III	30	164	74.00	27.50	n
8	SD	x		x		f	52	3520	34	39+4	VI	34	169	79.00	27.70	n
9	CS	x				f	53	4070	34	40+0	I	34	176	62.00	20.00	n
10	CS	x		x		f	51	2990	37	38+1	III	36	168	138.40	49.00	n
11	CS	x		x												
12	CS	x		x		m	51	3280	35	39+0	IV	35	156	54.00	22.20	n
13	SD	x		x		f	48	2920	33	38+4	II	33	171	102.00	34.90	n
14	CS	x		x		f	49	3100	34	39+0	III	40	160	55.00	21.50	n
15	SD	x				f	50	3750	36	39+1	II	28	164	70.00	26.00	
16	CS	x		x		m	49	3130	36	39+0	I	26	168	83.10	29.40	n
17	CS	x		x		m	50	3640	35	39+2	I	30	160	60.00	23.40	
18	CS	x		x		m	48	3580	36	39+3	IV	34	159	84.00	33.20	n
19	SD	x		x		m	50	3210	35	39+0	II	31	169	61.50	21.50	n
20	CS	x	x	x		m	50	3230	34.5	40+2	III/III	32	157	73.70	29.90	y
21	SD	x		x		m	51	3550	35	41+3	III/II	32	153	57.40	24.50	n
22	SD	x	x	x		f	52	3610	35	39+1	II/I	32	168	84.00	29.80	n
23	SD		x			m	53	3260	33	38+1	I/I	29	156	60.00	24.70	n
24	CS		x			m	51	3150	35	39+0	II/II	36	165	66.00	24.20	n
25	CS		x			m	51	3130	34	39+3	I/I	23	164	57.00	21.20	
26	CS		x			m	51	3700	37	39+0	II/II	34	163	77.90	29.30	n
27	CS		x			m	55	3650	37	39+0	III/II	35	170	99.00	34.30	n
28	SD		x			m	51	3890	37	39+3	I/I	31	176	82.00	26.50	
29	CS		x			m	48	3240		39+0	IV/III	26	154	60.00	25.30	
30	CS		x			m	51	3190	34	39+0	II/II	30	166	60.00	21.80	y
31	SD		x			m	51	3370	33	40+0	III/I	21	165	83.00	30.50	n
32	CS		x			f	50	2920	34	38+1	III/III	33	165	58.00	21.30	n
33	SD	x				m	50	3440	34.5	39+1	II/I	34	172	62.00	21.00	n
34	SD	x				m	53	3970	34.5	41+0	I/I	26	164	73.00	27.10	n
35	CS		x			f	50	3260	36	38+0	VI/IV	43	170	106.00	36.70	n
36	CS		x			m	49	2640	34	38+2	VI/VI	33	155	83.00	34.50	n
37	CS		x			f	48	2810	35	38+6	III/III	38	164	70.00	26.00	n
38	CS					m	48	3070	36	38+4	III/II	29	155	59.00	24.60	n
39	SD					m	50	3540	35.5	39+5	IV/III	36	157	75.00	30.40	n
40	SD		x			f	51	3550	35	41+2	II/II	36	167	53.70	19.30	n
41	SD		x			m	48	3260	35	38+0	II/II	31	165	60.30	22.10	n
42	CS		x			m	53	3360	36	38+0	I/I	42	170	61.00	21.10	n
43	SD		x			f	51	3910	37	40+3	IV/III	27	160	90.00	35.20	n
44	CS			x		m		2800		38+5	I/I	25	160	102.00	39.80	n
45	SD		x	x		f	47	2930	31	38+2	I/I	20	159	54.00	21.40	y
46	CS		x			f	51	3460	35	39+0	IV/II	27	168	88.00	31.20	n
47	SD		x			m	49	3110	34	40+4	III/II	27	165	85.00	31.20	n
48	CS		x			f	45	2860	34	38+2	III/II	37	160	48.00	18.80	n
49	CS		x			f	53	3930	34	38+4	V/IV	39	168	90.00	31.90	n
50	CS		x	x		f	52	3520	37	38+5	II/II	31	169	84.00	29.40	n
51	SD	x		x		f	49		35	38+4	IV/II	39	164	65.00	24.20	n
52	CS			x		m	51		37	39+1	III/I	34	164	115.00	42.80	n
53	SD	x		x		m	51		36	40+0	I/I	29	175	73.00	23.80	n
54	SD				x	f	46	2960	33.5	39+2	I	27	175	79	25.80	n
55	SD				x	m	50	3550	35	39+2	II	35	168	70	24.80	n
56	SD				x	m	50	3280	34	39+4	IV	39	160	47	18.40	n
57	SD				x	m	49	3110	35.5	40+3	II	29	157	79	32.00	y
58	SD				x	m	51	3630	36	41+1	I	24	165	59	21.70	n
59	CS				x	m	48	3350	36	38+2	III	31	155	90	37.50	n
60	CS				x	f	49	3020	34	39+1	III	27	164	61	22.70	n
61	CS				x	m	49	3940	36.5	39+1	III	36	165	49	18.00	n
62	CS				x	f	48	3420	35	38+5	XI	39	165	93	34.20	n
63	CS				x	f	46	2800	31	39+0	II	22	177	98	31.30	n
64	CS				x	f	48	3150	35	39+0	I	35	164	64	23.80	n
65	CS				x	f	52	4315	37	39+5	II	32	172	132	44.60	n
66	CS				x											

Supplementary Table S1: Clinical information on child and mother for all fetal membranes (FMs) included in the mechanical and structural analysis of the study. From left to right, the table shows the type of delivery ("SD" = spontaneous delivery, "CS" = cesarean section),

which methods were used to probe the respective sample (MRE, rheometer (“Rheo”), AFM, histological analysis (“Histo”)), information about the child (sex, height, weight, head size), the gestational age (“GA”) and information about the mother (parity, age, height, weight, BMI, smoking habits).
